# The effect of perceptual expectation on repetition suppression to faces is not modulated by variation in autistic traits

**DOI:** 10.1016/j.cortex.2015.10.011

**Published:** 2016-07

**Authors:** Michael P. Ewbank, Elisabeth A.H. von dem Hagen, Thomas E. Powell, Richard N. Henson, Andrew J. Calder

**Affiliations:** a Medical Research Council, Cognition and Brain Sciences Unit, Cambridge, UK; b School of Psychology, Cardiff University, Cardiff, UK

**Keywords:** Autism, Predictive-coding, Priors, fMRI-adaptation, Fusiform-face-area

## Abstract

There is substantial variation in the magnitude of the repetition suppression (RS) effects across individuals; however the causes of this variation remain unclear. In a recent study, we found that RS in occipitotemporal cortex was negatively related to individual variation in autistic traits in a neurotypical population. Recent proposals have considered autistic behaviours within a Bayesian framework, suggesting that individuals with autism may have ‘attenuated priors’ (i.e., their perception is less influenced by prior information). Predictive coding represents a neural instantiation of Bayesian inference, and characterises RS as reduction in prediction error between ‘top-down’ (prior beliefs) and ‘bottom-up’ (stimulus related) inputs. In accordance with this, evidence shows that RS is greater when repetition of a stimulus is expected relative to when it is unexpected. Here, using an established paradigm which manipulates the probability of stimulus repetition, we investigated the effect of perceptual expectation on RS in a group of neurotypical individuals varying on a measure of autistic traits. We predicted that the magnitude of the perceptual expectation effect would be negatively related to individual differences in autistic traits. We found a significant effect of perceptual expectation on RS in face-selective regions (i.e., greater RS when repetitions were expected relative to unexpected). However, there was no evidence of a relationship between autistic traits and the magnitude of this effect in any face-selective region of interest (ROI). These findings provide a challenge for the proposal that autism spectrum conditions (ASC) may be associated with the attenuated influence of prior information.

## 1 Introduction

Repetition of the same stimulus is associated with a reduction in BOLD response, known as fMRI-adaptation or repetition suppression (RS) (Grill-Spector and Malach, 2001, Henson, 2003). As with all physiological measures, there is substantial variation in the magnitude of the RS effect across individuals; however the causes of this variation remain unclear. In a recent study, we found that RS in category-selective regions of occipitotemporal cortex – coding faces, scenes or geometric shapes – showed a negative relationship with individual variation in autistic traits (Ewbank et al., 2014). Variation in autistic traits is proposed to constitute a continuum that extends from the neurotypical population to those with a clinical diagnosis of an autism spectrum condition (ASC) (Baron-Cohen, 1995), a neurodevelopmental condition associated with difficulties in social communication, narrow interests and repetitive behaviours (American Psychiatric Association, 2013).

An understanding of the implications of reduced RS in individuals with high numbers of autistic traits requires an understanding of the neural mechanisms that underlie this effect. A common interpretation is that RS is a ‘bottom-up’ effect, with reduced BOLD signal reflecting fatigue of a neuronal population responding to a particular stimulus, or a sparser encoding of the repeated stimulus [see Grill-Spector, Henson, & Martin (2006)]. According to predictive coding models – a neural instantiation of Bayesian inference – perception relies on matching top-down prediction signals (prior beliefs) from higher-order areas with sensory feed-forward signals. Thus, repetition of a stimulus leads to a reduction in neural activity in a given area because it reflects a decrease in prediction error between stimulus-related and prediction-related inputs (Friston, 2005, Henson, 2003). Consistent with the role of higher-level modulations in RS, we used Dynamic Causal Modelling to show that RS to faces or bodies in occipitotemporal cortex reflects changes in ‘top-down’ connectivity, with ‘higher-level’ regions modulating activity in ‘lower-level’ regions during repetition of the same body/face across changes in size/view (Ewbank et al., 2013, Ewbank et al., 2011).

Further support for the claim that RS is not a purely a ‘bottom-up’ mechanism, comes from a study by Summerfield, Trittschuh, Monti, Mesulam, and Egner (2008). They showed that RS to faces in the fusiform face area (FFA) (Kanwisher, McDermott, & Chun, 1997) was greater in blocks in which repetition of a face was more frequent (expected) than in blocks where repetition was less frequent (unexpected). A number of studies have since replicated the finding that RS to faces is modulated by stimulus repetition probability (Kovacs et al., 2013, Larsson and Smith, 2012, Summerfield et al., 2011), although the extent to which this effect generalises to non-human primates (Kaliukhovich & Vogels, 2011), or is dependent upon the type of stimulus used, remains unclear (Grotheer and Kovacs, 2014, Kovacs et al., 2013).

If predictive coding models of RS are correct, then individual differences in RS may reflect differences in intrinsic predictive processes. Reduced RS in individuals with high numbers of autistic traits would therefore accord with the proposal that perceptual atypicalities found in ASC can be explained as an attenuated influence of prior knowledge (Mitchell & Ropar, 2004). More recently, Pellicano and Burr (2012) provided a Bayesian formalization of this proposal, suggesting that perceptual atypicalities sometimes found in ASC, such as superior processing of embedded figures and reduced susceptibility to visual illusions (Simmons et al., 2009), might be a consequence of ‘attenuated priors’, suggesting previous experience has less influence on perception in ASC. Impairments in prediction have also been proposed to underlie other non-social and social symptoms found in autism (Sinha et al., 2014). Similarly, Lawson, Rees, and Friston (2014) recently proposed that many symptoms of autism can be explained within the Bayesian predictive coding framework as aberrant encoding of precision (i.e., an imbalance of the precision ascribed to sensory evidence relative to prior beliefs). Although both proposals emphasise that different aspects of Bayesian inference may be atypical in autism, both ‘attenuated priors’ and ‘aberrant encoding of precision’ theories have the same functional consequence for behaviour (i.e., perception is less sensitive to context).

Our previous work, showing reduced RS with increasing autistic traits (Ewbank et al., 2014), used a block-design format, where repetitions were always highly predictable. The aim of the current study was to use the Summerfield et al. (2008) paradigm to manipulate the probability of a repetition of a stimulus, and hence investigate the influence of perceptual expectation on the relationship between RS and autistic traits. We first expected to replicate the finding of greater RS to faces when stimulus repetitions were expected relative to when repetitions were unexpected (“perceptual expectation effect”). Secondly, if diminished RS as a function of autistic traits is a consequence of reduced use of prior information, we predict that the magnitude of the perceptual expectation effect would be negatively related to individual variation in autistic traits.

## 2 Methods and materials

### 2.1 Participants

Thirty-two neurotypical volunteers participated in the experiment. The data from three participants were removed due to excessive head movement in the scanner (>3 mm), leaving a total of 29 participants [16 female, all right-handed, aged 19–37 years old, mean age = 26.9 (SD = 6.0)]. Participants were recruited through the MRC Cognition and Brain Sciences Unit's research participation system. All participants had normal or corrected-to-normal vision. None had a history of head injury, neurological or psychiatric conditions (including autism), or was currently on medication affecting the central nervous system. The study was approved by the Cambridge Psychology Research Ethics Committee. All volunteers provided written informed written consent and were paid for participating.

### 2.2 Stimuli

For the localizer scan and RS experiment, we used a total of 572 black and white photographs of unfamiliar faces with neutral expressions (50% female). Images were obtained from the NimStim Face Stimulus Set (Tottenham et al., 2009), the Karolinska Directed Emotional Faces (KDEF) image set (Lundqvist & Litton, 1998), the FERET database (Phillips, Moon, Rizvim, & Rauss, 2000), the Psychological Image Collection at Stirling (PICS) (http://pics.psych.stir.ac.uk), and The Center for Vital Longevity Face Database (Minear & Park, 2004). All faces were cropped using an ovular mask. Different faces were used in the localiser scan and RS experiment.

### 2.3 Localizer scan

Participants lay supine in the magnet bore and viewed images projected onto a screen visible via an angled mirror. The localizer comprised images of 32 unfamiliar faces, 32 scenes, 32 household objects and 32 scrambled version of the objects. These were presented using a block design, consisting of four 16 sec blocks for each of the four conditions; each block contained 8 images with each image shown for 1600 msec followed by a 400 msec blank inter-stimulus interval (ISI). Blocks of stimuli were separated by an 8 sec rest block (fixation). To ensure participants were attending to all trials in the localizer scan they performed a target detection task and responded, via a button press, whenever they saw a green dot appear on an image (between one and two trials per block). Running time was approximately 12.5 min.

### 2.4 Repetition suppression experiment

**Fig. 1:**
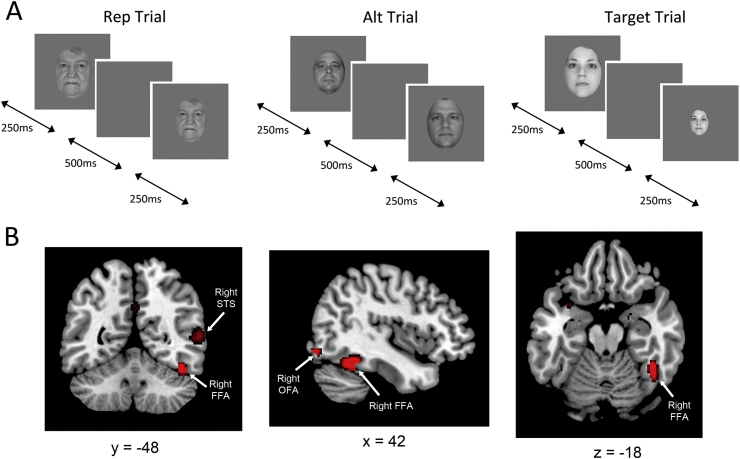
(A) Experimental format used in the RS experiment. An example of a repetition trial (Rep Trial), an alternation trial (Alt Trial), and a target trial are shown. Rep and Alt Trials appeared in blocks with a high probability (60%) of Rep Trials occurring (Rep Blocks) or in blocks with a low probability (20%) of Rep Trials occurring (Alt Blocks). 20% of trials were Target Trials. (B) Average face-selective FFA, OFA and STS across all participants identified with the localizer scan, shown (from left to right) on coronal, sagittal and transverse sections of an average T1 weighted image.

As in previous studies, we used a 2 × 2 mixed block/event-related design (Kaliukhovich and Vogels, 2011, Summerfield et al., 2008). Each trial comprised of two face stimuli, presented for 250 msec each, separated by an inter-stimulus interval of 500 msec, with a variable inter-trial interval between 1500 msec and 3000 msec. The first face stimulus was either identical to the second stimulus (Rep Trial) or different from the second stimulus (Alt Trial) (Fig. 1A). To reduce adaptation to low-level features, the size of images were varied by approximately 25% within each trial. Full size images subtended a visual angle of approximately 9° × 6°. The participants' task was to respond, via a button press, when they saw a 60% smaller image. In each block, 4 trials were target trials, which were either Alt Trials or Rep Trials with equal probability. Trial types were presented within the context of two different types of blocks (Summerfield et al., 2008). In the Repetition Blocks (Rep Block), 75% of non-target trials were Repetition Trials while 25% were Alternation Trials. In the Alternation Blocks (Alt Block), 75% of non-target trials were Alternation Trials and 25% were Repetition Trials. The first four trials of each block always consisted of the more frequent trial type of that block (Rep Trial in Rep Block and Alt Trial in Alt Block), while Rep Trials and Alt Trials were presented randomly within the rest of the block. There were a total of 18 blocks (9 Alt Blocks; 9 Rep Blocks) each contained 20 trials, making a total of 108 Rep Trials in Rep Blocks; 108 Alt Trials in Alt Blocks; 36 Rep Trials in Alt Blocks; 36 Alt Trials in Rep Blocks. Blocks were separated by a 2000 msec interval during which the words “Start of new block” appeared on screen. Rep and Alt blocks were alternated throughout the experiment. Running time was approximately 24.5 min.

Prior to scanning, participants completed the Autism-Spectrum Quotient (AQ) questionnaire, a 50-item validated measure of autistic traits that is suitable for use with neurotypical participants (Baron-Cohen, Wheelwright, Skinner, Martin, & Clubley, 2001); higher scores indicate increased numbers of autistic traits. Mean AQ scores = 15.9(SD = 9.0), range 4–37. Only three participants scored greater than 32, a level above which 79% of individuals with high functioning autism/Asperger syndrome scored in a previous study (Baron-Cohen et al., 2001). However, the AQ is not a diagnostic measure (Baron-Cohen et al., 2001) and no participants had a clinical diagnosis of an ASC.

### 2.5 Imaging parameters

MRI scanning was performed on a Siemens Tim Trio 3-T MR scanner with a 32-channel head coil. Brain data were acquired with T2*-weighted echo-planar imaging (EPI) sensitive to BOLD signal contrast (32, 3 mm thick slices; gap 25%; FOV 192 × 192 mm; flip angle 78°; TE 30 msec; TR 2 sec). Slices were acquired sequentially in an axial orientation aligned along the ventral temporal lobes. The first 3 volumes were discarded to allow for the effects of magnetic saturation. A high-resolution structural magnetization prepared rapid gradient echo scan was also acquired at a resolution of 1 × 1 × 1 mm.

### 2.6 fMRI analysis

Data were analysed using SPM 8 software (Wellcome Trust Centre for Neuroimaging, London, UK). For both the localiser and RS scan, standard pre-processing was applied, including correction for slice-timing and head motion. Each participant's scans were normalized using the linear and nonlinear normalization parameters estimated from warping the participant's structural image to the Montreal Neurological Institute (MNI) – ICBM avg152 T1 weighted template, using 2 mm isotropic voxels and smoothed with a Gaussian kernel of 8 mm full-width half-maximum. For the RS scan, a general linear model was created containing separate regressors for each of the four experimental conditions (Rep Trials in Rep Blocks; Alt Trials in Rep Blocks; Rep Trials in Alt Blocks; Alt Trials in Alt Blocks). Regressors were also included for target trials, and ‘New Block’ stimulus events. Regressors were convolved with a canonical hemodynamic response function (HRF). For the localizer scan, regressors for each condition were modeled by sustained epochs of neural activity (boxcars) convolved with a HRF. Finally, realignment parameters were included as effects of no interest to account for motion-related variance, and a high pass filter of 128 sec was used to remove low-frequency noise.

#### 2.6.1 Region of interest (ROI) analysis

Using the localizer scan, face-selective ROIs (faces > scenes) were identified in each participant at a minimal threshold of *p* < .01 uncorrected (10 contiguous voxels). Mean parameter estimates for each condition were then extracted from an 8 mm radius sphere centered on the maximal voxel in each participant's left and right face-selective occipital face area (OFA), FFA and right superior temporal sulcus (STS) using MarsBar (Brett, Anton, Valabregue, & Poline, 2002). Using SPSS version 22 (IBM Corp.), parameter estimates extracted from each ROI were entered into ANCOVAs including Trial (Rep, Alt), and Block (Rep, Alt) as repeated measures factors, sex as a between participants' factor, and mean centred AQ scores and age included as a covariates.

Note that the analysis of the RS experiment was conducted on data extracted from ROIs that were independently defined using localizer scans. Hence, voxel selection was blind to any possible relationship between these voxels and AQ, avoiding the logical and statistical biases that may lead to inflated correlations (Calder et al., 2011, Vul et al., 2009).

#### 2.6.2 Whole brain analysis

To determine whether perceptual expectation effects were found in regions outside face-selective ROIs, we performed an exploratory whole-brain analysis including all 29 participants. For each participant, first-level images of parameter estimates for each of the four conditions (Rep Trials in Rep Blocks; Alt Trials in Rep Blocks; Rep Trials in Alt Blocks; Alt Trials in Alt Blocks) were entered into a repeated measures 2 × 2 ANOVA examining the effects of Trial (Rep Trial, Alt Trial) and Block (Rep Block, Alt Block) (*p* < .05 FWE corrected, 10 contiguous voxels). Finally, to determine whether any regions outside of the face-selective ROIs showed a relationship between perceptual expectation and AQ, for all 29 participants, first-level contrast images of the interaction between Trial and Block [(Alt Trial Rep Block > Rep Trial Rep Block) > (Alt Trial Alt Block > Rep Trial Alt Block)] (i.e., perceptual expectation effect) were entered into a whole brain, group level regression analysis with AQ and age as covariates (*p* < .05 FWE corrected, 10 contiguous voxels).

## 3 Results

### 3.1 Localizer scan

Using the contrast faces > scenes, in the right hemisphere we localized FFA in 26 out of 29 participants, OFA in 22 participants and STS in 19 participants (Fig. 1B). In the left hemisphere, FFA was identified in 23 participants and OFA in 17 participants. Mean (SD) MNI coordinates for each ROI: Right FFA: +41(3.0), −48(4.4), −19(4.3); Left FFA: −39(2.6), −50(8.3), −18(3.8); Right OFA: +42(6.1), −78(7.3), −9(4.5); Left OFA: −42(3.9), −76(8.3), −11(6.0); Right STS: +55(6.4), −48(9.5), +9(5.4).

### 3.2 Repetition suppression experiment

#### 3.2.1 ROI analysis

To examine the influence of AQ on the perceptual expectation effect, data extracted from each ROI were entered into separate ANCOVAs examining the effects of Trial (Rep Trial, Alt Trial) and Block (Rep Block, Alt Block) as repeated measures factors. As in previous work, sex was included as a between participants' factor, with AQ scores and age entered as covariates (Ewbank et al., 2014). ANCOVAs revealed no evidence of a main effect of AQ in any ROI (*p*'s > .11) suggesting that the overall response to faces did not differ as a function of AQ scores.

**Fig. 2:**
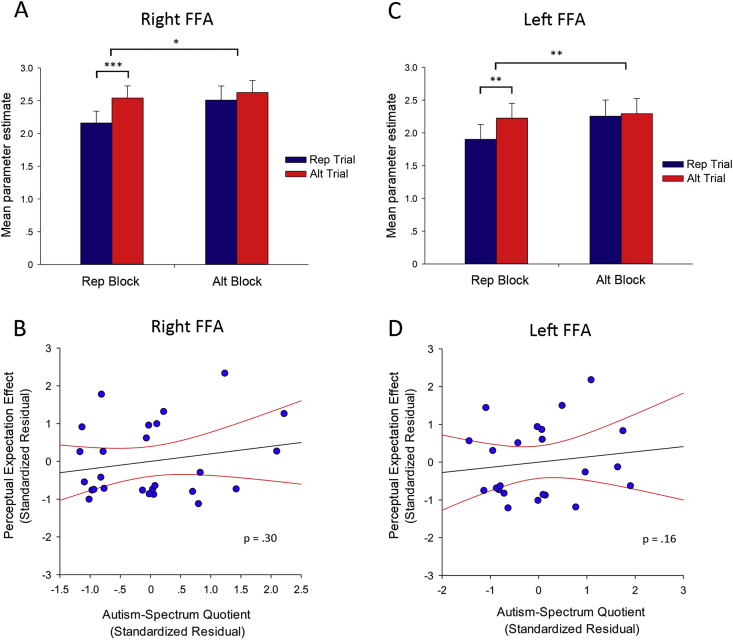
(A) Mean parameter estimates (+1SD) for all conditions (Rep Trials or Alt Trials appearing in Rep Blocks or Alt Blocks) in right FFA and (C) left FFA. Upper horizontal bar represents interaction between Trial and Block. (B) Relationship between Autism-Spectrum Quotient (AQ) and perceptual expectation effect in right FFA and (D) left FFA. All scatter plots show standardized residuals of contrast estimates of the perceptual expectation effect (covarying out effects of age) plotted against standard residuals of individual AQ scores. Regression line and 95% confidence intervals are shown. *p < .05, **p < .005, ***p < .001.

For the right FFA, the ANCOVA revealed a significant effect of Trial (i.e., a greater response to Alt Trials compared to Rep Trials) [*F*(1,22) = 12.92, *p* < .005, *ηρ*^2^ = .37]. In addition, there was a significant interaction between Trial and Block [*F*(1,22) = 6.41, *p* < .05, *ηρ*^2^ = .23] (Fig. 2A). Paired comparisons revealed a significantly greater response to Alt Trials compared to Rep Trials when Rep Trials occurred with a high probability [t(25) = 4.61, *p* < .001] but not when occurring with a low probability [t(25) = 1.04, *p* = .17] (perceptual expectation effect). However, we found no evidence of a significant interaction between Trial, Block and AQ (*p* = .31) (Fig. 2B), and no interactions between Trial or Block and AQ (*p*'s > .41), indicating that the magnitude of the perceptual expectation effect in right FFA was not modulated by AQ scores.

In left FFA, an analogous ANCOVA revealed a borderline effect of Trial (*p* = .12), with a trend towards a greater response in the Alt Trials compared to the Rep Trials, and a main effect of Block [*F*(1,19) = 6.39, *p* < .05, *ηρ*^2^ = .25], with a greater response in Alt Blocks relative to Rep Blocks. This effect was qualified by a significant interaction between Trial and Block [*F*(1,19) = 14.85, *p* < .005, *ηρ*^2^ = .44] (Fig. 2C). Paired comparisons revealed a significant difference between Alt and Rep Trials in Rep Blocks [t(22) = 4.04, *p* < .005] but not in Alt Blocks [t(22) = .49, *p* = .63]. Again, we found no evidence of any significant interactions involving Trial and/or Block and AQ (*p*'s > .16) (Fig. 2D).

**Fig. 3:**
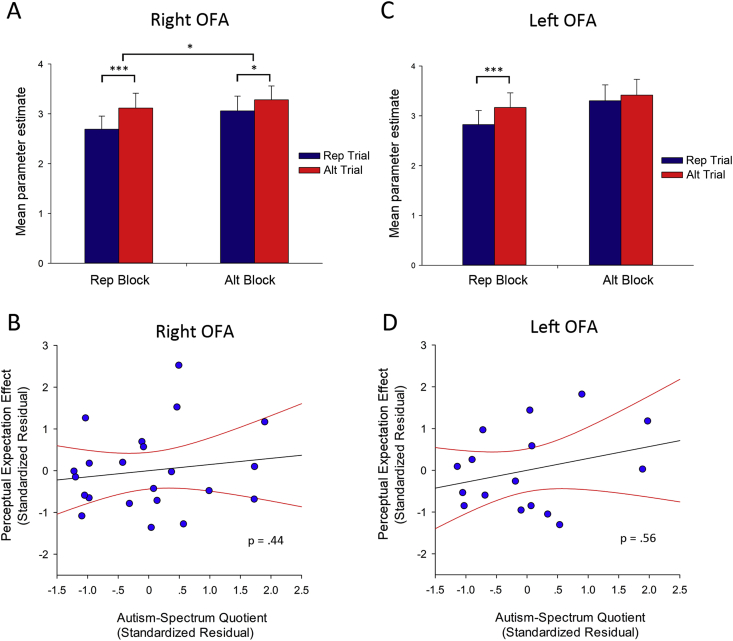
(A) Mean parameter estimates (+1SD) for all conditions (Rep Trials or Alt Trials appearing in Rep Blocks or Alt Blocks) in right OFA and (C) left OFA. Upper horizontal bar represents interaction between Trial and Block. (B) Relationship between Autism-Spectrum Quotient (AQ) and the perceptual expectation effect right OFA and (D) left OFA. All scatter plots show standardized residuals of contrast estimates of the perceptual expectation effect (covarying out effects of age) plotted against standard residuals of individual AQ scores. Regression line and 95% confidence intervals are shown. *p < .05, ***p < .001.

For the right OFA, there was a significant effect of Trial [*F*(1,18) = 33.84, *p* < .001, *ηρ*^2^ = .65], reflecting a greater response in Alt Trials, and a significant effect of Block [*F*(1,18) = 8.07, *p* < .05, *ηρ*^2^ = .31], reflecting a greater overall response in Alt Blocks (Fig. 3A). This effect was again qualified by an interaction between Trial and Block [*F*(1,18) = 5.59, *p* < .05, *ηρ*^2^ = .24] reflecting a greater difference between Alt and Rep Trials in Rep Blocks [t(21) = 5.26, *p* < .001] than in Alt Blocks [t(21) = 2.35, *p* < .05]. Crucially there was no interaction between Trial, Block and AQ (*p* = .45) (Fig. 3B), nor any other interaction involving AQ in this region (*p*'s > .17).

In left OFA, the ANCOVA revealed a main effect of Trial [*F*(1,13) = 5.84, *p* < .05, *ηρ*^2^ = .31], reflecting a greater response in Alt Trials, and a main effect of Block [*F*(1,13) = 8.13, *p* < .05, *ηρ*^2^ = .39], reflecting a greater response in Alt Blocks, and a non-significant trend towards an interaction between Trial and Block (*p* = .09) (Fig. 3C). There was a significant interaction between Block and AQ [*F*(1,13) = 4.63, *p* = .05, *ηρ*^2^ = .26], reflecting a reduced response in Alt Blocks as a function of increasing AQ, but crucially no significant interaction between Trial, Block and AQ (*p* = .46), indicating that the perceptual expectation effect was not modulated by AQ scores (Fig. 3D). In right STS there were no main effects (*p*'s > .11), and no evidence of any interactions involving Trial and/or Block and AQ (*p*'s > .32). Finally, there were no main effects or interactions involving either sex (*p*'s > .17) or age (*p*'s > .08) in any region.

#### 3.2.2 Whole brain analysis

An exploratory whole brain analysis revealed no main effects of Trial or Block and no interaction between Trial and Block that survived correction for multiple comparisons (*p* < .05 FWE). At a more liberal threshold (*p* < .001 uncorrected), there was a main effect of Trial in regions corresponding to right and left FFA and right OFA, and also a main effect of block in these regions (Table 1). No regions showed a perceptual expectation effect (i.e., a significant interaction between Trial and Block) at a whole brain corrected level (*p* < .05 FWE) or even at a more liberal threshold (*p* < .001 uncorrected). A regression analysis, with AQ as a covariate, revealed no evidence of a significant negative relationship between AQ scores and perceptual expectation at a whole brain corrected level (*p* < .05 FWE) or at a more liberal threshold (*p* < .001 uncorrected). Regions showing a positive correlation between perceptual expectation and AQ at an uncorrected level (*p* < .001) are listed in Table 1.

#### 3.2.3 Behavioural data

When questioned at the end of the experiment, none of the participants reported any awareness of the different repetition probabilities in different blocks. A paired *t*-test revealed that accuracy rates for the size change-detection task did not differ between Alt and Rep Trials (*p* = .89) [mean (SE) = Rep Trial: 96.6% (1.2); Alt Trial: 96.7% (1.6)] or Alt and Rep Blocks (*p* = .69) [mean (SE) = Rep Block: 96.2% (1.4); Alt Block: 96.8 (1.5)]. Similarly, there was no difference in reaction times (RT's) between Alt and Rep Trials (*p* = .65) [mean (SE) = Rep Trials: 521.1 (10.8); Alt Trial: 519.9 (12.3)] or between Alt and Rep Blocks (*p* = .10) [mean (SE) = Rep Block: 527.4 (14.1); Alt Block: 539.2 (14.2)]. Finally, a Pearson's correlation analysis revealed no evidence of a significant relationship between AQ and accuracy or AQ and RTs for either Rep Trials or Alt Trials or Rep Blocks or Alt Blocks (r's < .15).

## 4 Discussion

The aim of this study was to investigate whether RS to faces is modulated by perceptual expectation, and to determine whether the magnitude of this effect is diminished as a function of increasing autistic traits. Using a paradigm that manipulates the probability of stimulus repetition (Summerfield et al., 2008), we found greater RS in face-selective regions during blocks with a high probability of repetitions relative to blocks with a low probability of repetitions (i.e., greater RS for expected relative to unexpected repetitions). However, we found no evidence that the magnitude of this effect was related to autistic traits in any face-selective region. This finding appears inconsistent with the proposal that attenuated influence of prior information may underlie reduced RS in individuals with higher numbers of autistic traits; instead suggesting that perceptual expectation modulates the magnitude of RS independent of individual variation in AQ.

The finding of greater RS to faces in right FFA during blocks with a high probability of repetition relative to blocks with a low probability of repetition is consistent with that of a number of other studies (Kovacs et al., 2012, Kovacs et al., 2013, Larsson and Smith, 2012, Summerfield et al., 2008). This result accords with a predictive coding account of RS, proposing that RS reflects a decrease in prediction error that occurs when expected and observed sensory information coincide, and provides a challenge for ‘bottom-up’ models of RS, such as neuronal fatigue. In our study, the effect of perceptual expectation was restricted to face-selective bilateral FFA and right OFA, with no effects found outside these areas. Note, some previous studies have reported that the effect of perceptual expectation on RS is due to an enhanced response to rare (or surprising) events (i.e., an increased response in Alt Trials in Rep Blocks relative to Alt Blocks) rather than a decreased response to fulfilled expectations (i.e., a reduced response in Rep Trials in Rep Blocks relative to Alt Blocks) (Grotheer and Kovacs, 2014, Mayrhauser et al., 2014). However, in the current study the effect of perceptual expectation in FFA reflected a decreased response to expected stimuli (i.e., greater suppression for Rep Trials in Rep Blocks) (see Fig. 2), and therefore our results accord with a model of a ‘prediction-related modulation’ rather than ‘surprise-related modulation’ [see Kovacs & Vogels (2014)]. Although a main effect of block (i.e., greater overall response in Alt Blocks), has not been reported in previous studies, we believe this finding is consistent with ‘fulfilled expectations’, given that it is driven by an increased response to unexpected Rep Trials relative to expected Rep Trials (Alt trials did not differ between blocks). One possibility is that this finding is a consequence of the greater statistical power of the current study (*n* = 29). Previous studies using the same paradigm to investigate the effects of expectation on RS to faces had sample sizes ranging between 8 and 16.

The absence of a negative relationship between perceptual expectation effects and AQ appears inconsistent with proposals that ASC is associated with an attenuated influence of prior experience on perception (Lawson et al., 2014, Pellicano and Burr, 2012). We believe the absence of a relationship with autistic traits is unlikely to be due to a restricted range of AQ scores in the current study, given that range of scores (4–37) was comparable to that of previous studies (Ewbank et al., 2014). Indeed, if anything, rather than a negative relationship, there was a non-significant trend towards an increase in perceptual expectation effects as a function of AQ in both FFA and OFA. An exploratory whole brain analysis also revealed a significant positive correlation between perceptual expectation and AQ in regions of medial prefrontal cortex (Table 1), suggesting that high numbers of autistic traits may be associated with increased activation in ‘higher-level’ cortical regions as a function of expectation. However, we are hesitant to make any strong inferences on the basis of these findings, given that we had no a priori hypothesis regarding activation outside of occipitotemporal cortex, and that an uncorrected threshold is generally deemed inappropriate when reporting a whole brain analysis (Poldrack, 2012, Poldrack et al., 2008).

It is possible that evidence for attenuated use of prior experience on RS is only found in individuals with a clinical diagnosis of autism and/or that any attenuation in the use of prior information is too small to be detected in neurotypicals using this paradigm. The advantage of investigating the effect of autistic traits in neurotypical participants is that it provides a complementary approach to studies of ASC, without any associated complications of clinical comorbidity (Leyfer et al., 2006). However, in order to fully investigate perceptual expectation and RS in autism, it will be necessary to address this question in individuals with a clinical diagnosis of ASC. In addition, without the inclusion of a neutral (50% probability) condition, this paradigm only provides a relative measure of the difference between neural activity due to fulfilled expectations and neural activity due to violated expectations (Kovacs & Vogels, 2014). Thus, it is unclear whether high and low AQ participants show the same relative increase and decrease in neural activity, relative to a baseline condition, for unexpected and expected events respectively.

The extent to which the absence of a relationship between AQ and the effect of perceptual expectation can be taken as evidence of intact predictive mechanisms in individuals with high autistic traits/autism, is of course, dependent upon the extent to which the paradigm used here is a true measure of predictive coding. It is important to consider that the ‘predictions’ addressed in predictive coding are described as a “prediction of sensory effects from their causes … and are not about predicting sensory states in the future, given the sensory state now” (Kilner, Friston, & Frith, 2007). In this sense, the current paradigm may more accurately be said to reflect a test of ‘prospective coding’ (Rainer, Rao, & Miller, 1999). Moreover, the ‘predictions’ in predictive coding are characterised as automatic, intrinsic properties of cortical networks and do not depend on conscious expectation (Friston, 2005). Indeed, MEG evidence suggests that RS to stimulus repetition and stimulus expectation may be dissociable; repetition leading to RS of an early auditory component (40–60 msec) and expectation associated with RS at a later time period (100–200 msec) (Todorovic & de Lange, 2012). The extent to which the ‘predictions’ manipulated in the current paradigm are conscious is unclear (although it should be noted that no participant reported any awareness of the different repetition probabilities in different blocks). We also note that Larsson and Smith (2012) found that effects of perceptual expectation on RS were dependent upon attention being directed to the stimulus. Thus, enhanced RS in high Rep Blocks might be due to infrequent/novel trials capturing attention (i.e., Alt Trials in Rep Blocks and Rep Trials in Alt Blocks). Although in the current study, unexpected Alt Trials did not evoke a greater signal than expected Alt Trials, which appears inconsistent with an entirely attentional based explanation. Furthermore, the extent to which attention can be dissociated from prediction error remains unclear (Feldman & Friston, 2010).

In previous work, we have shown that the magnitude of RS to faces in the FFA of neurotypical participants is negatively related to individual differences in autistic traits (an effect replicated across two groups of participants) (Ewbank et al., 2014). Moreover, this relationship was found to extend to RS to images of scenes and simple geometric shapes in scene- and object-selective regions of occipitotemporal cortex respectively. However, in the current study we found no evidence of a relationship between AQ and overall RS effects (i.e., all Alt Trials *vs* all Rep Trials). Although this result appears to contrast with the findings of the previous study, it is difficult to assume equivalence between the repetition effects found in the two studies. First, repetitions in the current study always appeared in a specific context – reflecting low or high probability of a Rep Trial (25% or 75%) – whereas the previous study presented stimuli in blocks where all faces were the same/different identity (100% probability of repetition/alternation). Second, in a block design, repetitions are highly predictable and consciously expected, whereas in the current study participants did not report any awareness of the likelihood of repetitions. It is therefore interesting to consider whether any difference in the relationship between AQ and RS between studies reflects differences in the use of explicit and implicit predictions between high and low AQ participants. Indeed, behavioural studies suggest that implicit learning appears generally unimpaired in individuals with high functioning autism [see Boucher, Mayes, & Bigham (2012)]. Future work will be needed to directly compare the relationship between AQ/autism and RS using both a block and event-related paradigm (where participants have few or no expectations about the next stimulus) in the same group of participants.

In conclusion, using a paradigm that manipulated the probability of stimulus repetition, we found greater RS to faces in blocks where repetitions were expected relative to blocks where repetitions were unexpected. This result is consistent with that reported by a number of previous studies and suggests a role for ‘higher-level’ predictions in modulating RS. Based on previous work, we predicted that the magnitude of this effect would be negatively related to individual variation in autistic traits. The results revealed no evidence of a relationship between autistic traits and the effect of perceptual expectation on RS. These findings appear inconsistent with the idea of attenuated influence of prior information in ASC.

## Figures and Tables

**Table 1:** MNI coordinates of brain regions showing a significant main of effect of Trial (Alt > Rep), a significant main effect of Block (Alt > Rep), and a positive correlation between AQ scores and perceptual expectation [(Alt Trial Rep Block > Rep Trial Rep Block) > (Alt Trial Alt Block > Rep Trial Alt Block)] (covarying out effects of age). All activations significant at *p* < .001 uncorrected (10 contiguous voxels) at the whole-brain level.

Brain region	Hemisphere	X	Y	Z	Cluster size	T
***Main effect of Trial* (*Alt* > *Rep*)**						
Mid fusiform gyrus	R	36	−48	−20	112	4.02
	L	−38	−50	−10	95	3.69
Inferior occipital gyrus	R	40	−80	−12	171	3.93
***Main effect of Block* (*Alt* > *Rep*)**						
Mid fusiform gyrus	L	−32	−52	−12	77	4.65
	R	36	−46	−12	35	3.90
Posterior fusiform gyrus	R	34	−60	−8	94	3.96
Cuneus	R	16	−72	6	30	3.74
Inferior temporal gyrus	L	−40	−74	−2	31	3.73
Postcentral gyrus	R	52	−18	56	15	3.56
Insula	R	32	−2	14	14	3.51
***Positive correlation between AQ and perceptual expectation***						
Middle frontal gyrus	R	22	44	4	116	6.54
	L	−26	44	4	20	4.19
Superior frontal gyrus	R	20	−24	50	97	4.36
Inferior parietal lobule	R	44	−54	30	98	4.34
Medial frontal gyrus	R	10	48	−18	29	4.05
